# 

**Published:** 1894-03

**Authors:** 


### CONTENTS.

ORIGINAL COMMUNICATIONS
The Nature and Symptoms of Exophthalmic Goitre. By M. Le Docteur A. Joffroy, Paris, France .......... 1
The Essential Elements of Ideal Surgery. By B. F. Wilson, A. M., M. D., Slater, Missouri .......... 7
Treatment of Uterine Fibroids. By Augustin H. Goelet, M.D., New York .......... 10
Epsom Salts for the Pain in Burns. By N. F. Howard, M.D., Dahlonega, Ga .......... 13
Beechwood Creosote in Treatment of Phthisis. By Wm. J. Cox, M.D., Flovilla, Ga. .......... 14
SOCIETY REPORTS—
Clinical Society of Maryland .......... 16
Atlanta Society of Medicine .......... 21
CORRESPONDENCE—
New York Letter .......... 26
EDITORIAL—
The Castration of Women .......... 33
Medical Legislation in Ohio .......... 36
The Visit of the Medical Editors .......... 37
SELECTIONS AND ABSTRACTS—
Practical Chemistry and Pharmacy .......... 40
Surgery .......... 43
Gynecology and Obstetrics .......... 49
Skin Diseases and Syphilis .......... 54
General Medicine and Therapeutics .......... 56
MEDICAL ITEMS .......... 59
BOOK REVIEWS .......... 60
ADVERTISERS' INDEX TO ADVERTISEMENTS .......... xii
CLASSIFIED INDEX TO ADVERTISEMENTS .......... xxxvii
ITEMS OF INTEREST .......... x, xi
JOURNAL FOR 1894 .......... xxii
PREMIUM OFFERS .......... xxxvi

